# A Critical Interpretive Review of the Theoretical Literature Related to Healthcare Codes of Ethics

**DOI:** 10.1007/s11673-025-10452-5

**Published:** 2025-08-07

**Authors:** Ryan Essex, Lydia Mainey, Francine Gonzales-Walters, Phil Gurnett, Sharon Marie Weldon

**Affiliations:** 1 The George Institute for Global Health, New South Wales, Sydney Australia; 2 School of Population Health, University of New South Wales, Sydney, Australia; 3 School of Nursing, Midwifery and Social Sciences, Central Queensland University, Norman Gardens, Australia; 4 School of Health Sciences, University of Greenwich, London, UK; 5 Institute for Lifecourse Development, University of Greenwich, London, UK; 6 Barts Health NHS Trust, London, UK; 7 Imperial College London, London, UK

**Keywords:** Codes of ethics, Clinical ethics, Decision-making, Literature review

## Abstract

Codes of ethics have a long history in healthcare and, for many, are important documents. Codes however have also been extensively criticized for a range of reasons, from the guidance they provide to their meta-ethical assumptions. This review sought to explore the theoretical literature to critically examine the function of codes in healthcare, with a particular focus on their strengths and shortcomings in relation to these functions. A systematic search was combined with a critical interpretive review. The final sample included twenty-four papers. Results of this synthesis suggest that codes fulfil multiple purposes, from providing guidance on ethical issues, to assertions about acceptable and unacceptable behaviour, to establishing and maintaining the status and identity of the professions. Codes also fulfil a number of social purposes, conveying information to the public and others. The extent to which a code does each of these things varies substantially however. We discuss these functions in relation to the many critiques that have been advanced in relation to these documents. We then put these findings into conversation with the broader literature on codes and discuss the challenges that this presents for normative analysis, namely in needing to first identify what a code *should* do before assessing its shortcomings. If the primary purpose of a code of ethics is to provide guidance, many fail here, the devil is in the detail however. To what extent should codes provide guidance?

## Introduction

Codes of ethics have a long history in healthcare. What was arguably the first modern code, was proposed by Thomas Percivall, an English physician, in 1803. The code was developed in response to a conflict in the hospital in which he worked (Baker 2012). Since this time codes have proliferated and taken a range of forms, with international codes, codes for sub-specialities, and more general codes for the various health professions across the globe.

There is little doubt that codes are important to the healthcare professions.^1^ This is perhaps best reflected by some of the statements found in codes themselves. The code produced by the National Federation of Nurses in Belgium (2017) for example, states

“[t]his code specifies the values and standards which form the basis of nursing practice and which allow practitioners of the nursing art to practice their profession, in all situations, correctly and responsibly” (1).

The code produced by the Israeli Medical Association (2018, 0) states that [t]he ethical code constitutes a social covenant, one of the important foundations of society. On the one hand, society places its trust in its physicians that they will devote their capabilities, knowledge, and information to the benefit of individuals, while on the other hand the physician undertakes to observe the ethical code formulated by his professional colleagues.

The purpose of codes however is more difficult to pin down. We can again look to codes themselves for some guidance. There is little doubt that codes are written for those within the profession, with many dedicating space to discussing how a code should shape conduct and be interpreted. The American Medical Association (2016 clarifies that their principles of medical ethics “are not laws, but standards of conduct that define the essentials of honorable behavior for physicians.” The International Council of Nurses (2021, 4) code stresses that “[t]o achieve its purpose the Code must be understood, internalised and used by nurses in all aspects of their work.” Codes however are also directed to the public, expressing what standards they should expect when interacting with members of the profession. The Japanese Nursing Association (2003, 1) code of ethics for example, makes clear, that the code “specifies to society the scope of the responsibilities that nurses should take as professionals in practicing nursing.” The Medical College of Chile (2022) asserts that the promotion of ethical principles as they relate to the regulation of medicine not only serves to orient medicine to human well-being but also acts as a “beacon to society.” Beyond this however, codes somewhat diverge. Some give the impression they are comprehensive, while others call for the use of professional judgment in decision-making (South African Nursing Council 2020). Some explicitly state they are living documents, dynamic and changing in line with social and political realities (Danish Council of Nursing Ethics 2014). Some acknowledge their theoretical foundations and development (Malaysian Medical Association 2001), while others do not. Some are more explicit about the purpose that codes serve in relation to the profession, namely in relation to safeguarding the reputation of the profession in question (Danish Council of Nursing Ethics 2014).

There has of course been much said about the nature and function of codes. A common defence relates to codes being critical for the regulation and behaviour of healthcare professionals. Even if codes cannot provide guidance across all circumstances, they provide standards against which behaviour can be assessed, defining what the public should expect from the profession in question. Critics however have advanced arguments along similar lines, not only about the failure to guide behaviour but as documents that are self-serving and a means for the professions to entrench existing powers (Baker 2012). One recent review that sought to shed light on the question of codes’ function and content examined comparative studies of codes (i.e. studies that compared two or more codes) found that factors such as culture, history, politics, and even geography were influential in determining the content of codes (Essex, et al 2025). Notably, a scoping review that examined the use and knowledge of health professionals as it related to codes, it was found that most health professionals did not use their codes regularly and knew little about the content in their respective codes (Collings-Hughes et al. 2022).

In saying this, whatever it is that codes do, it is clear they matter, perhaps the more difficult questions however are to what extent, in what ways and for whom they matter. Regardless, it seems reasonable to suggest that codes are likely to remain a feature of the health professions into the foreseeable future. This study sought to critically examine the function of codes in healthcare, with a particular focus on their strengths and shortcomings of fulfilling these various functions.

## Methods

### Design

This paper employed a systematic search with a critical interpretive synthesis. This approach has been outlined by Dixon-Woods et al. (2006) and adapted for bioethics by McDougall (2015). A critical interpretive synthesis was particularly well suited to the above research question as it lends itself to the development of concepts and theory, utilizing both induction and interpretation in the synthesis of data (Dixon-Woods et al. 2006). This type of review has been used in a number of health disciplines including bioethics to answer theoretical, conceptual, and normative questions. For those familiar with systematic searches of the literature, our search, detailed below, follows PRISMA guidelines and is reported in a PRISMA flowchart (Page et al. 2021). The point of divergence for this review relates to the synthesis of the evidence from this search. In most traditional systematic or scoping reviews, all papers would be retained, at least until quality or bias was assessed. This is generally not the case for critical interpretive syntheses. This is because of the distinct nature of the research questions in such reviews, which are not concerned with effectiveness, for example. The research question above differs in that it relates to the conceptual and normative foundations of codes. For such a question (and others like it) it is not necessary and arguably undesirable to carry out an exhaustive synthesis of the literature. In other words, the above research question does not rely on data the same way that more traditional reviews do, nor does it require all papers on any given issue to be assembled for it to be answered (McDougall 2015). Consistent with this approach this review took the following steps: 1) development and framing of the research question 2) literature search and selection 3) data extraction and 4) data synthesis. Each of these steps is expanded upon below.

### Search Strategy

While a critical interpretive synthesis generally allows a degree of flexibility in terms of search strategy, a structured systematic approach served the needs of this study, providing a substantial sample of papers. Search terms were developed to capture the two key concepts, namely codes of ethics and the healthcare professions. The final search terms (or string) were (“code of ethics” OR “code of conduct”^2^) AND (doctor OR physician OR clinician OR “medical practitioner” OR nurs* OR “health profession*” OR healthcare OR “health care” OR “pharmac*” OR “dentist” OR “midwi*” OR dieti* OR “occupational therap*” OR “paramed*” OR “physiotherap*” OR “radiograph*” OR “psycholog*” OR “health worker” OR “hospital”). The search was executed on February 21, 2024 using Scopus, Medline, CINAHL, and PsycInfo. The reference lists of included papers were also searched for relevant articles.

### Search Results and Literature Selection

**Fig. 1:**
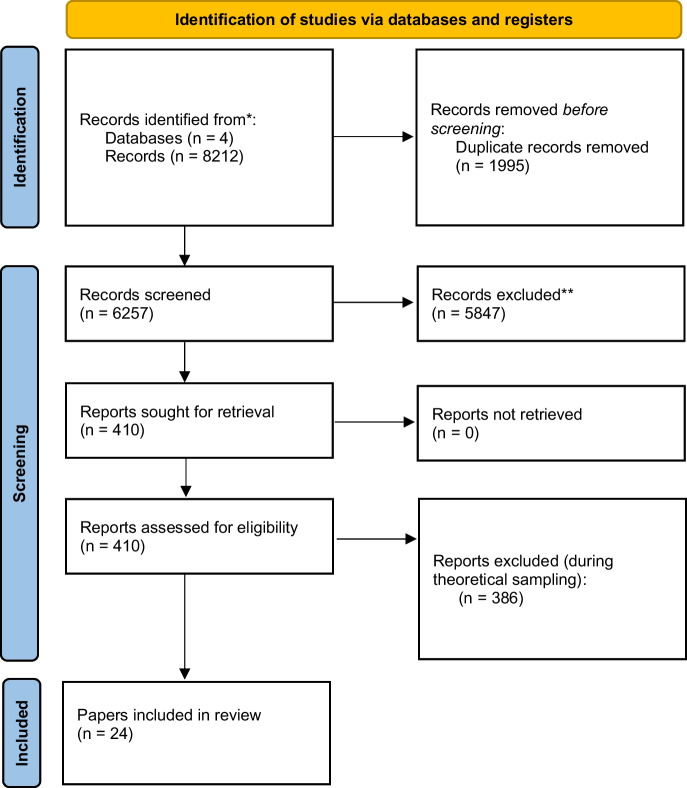
PRISMA flow diagram

The above search yielded 8212 results, with 6257 after duplicates were removed. A title and abstract screen was carried out, which left a pool of 410 publications. In these first two stages papers were screened in line with broad eligibility criteria, that is, papers were theoretical and discussed healthcare code of ethics. We excluded literature reviews and papers that were not written in English. At this stage, each paper was screened twice to ensure consistency in decision-making and that potentially eligible papers had not been overlooked. From the remaining pool of 410 publications, papers were selected based on whether they made a substantial contribution to understanding codes of ethics, usually related to the function of codes, their strengths, or shortcomings. These papers often contained substantial normative discussion or advanced a unique perspective related to codes of ethics. Many of the articles excluded at this point were shorter articles, that offered little new or novel reasoning related to codes, their function, strengths, or shortcomings. This left us with twenty-four papers. The results of this process are summarized in Figure 1.

While it is not necessary to carry out a quality appraisal in a critical interpretive synthesis, it is briefly worth mentioning some reflections on the above process in light of the quality of included papers. While more traditional systematic reviews conduct a quality appraisal for each of the included papers, such an approach presents difficulties in bioethics (McDougall 2015). In short, even where an “objective” criteria could be established, any quality appraisal would be vastly time-consuming and particularly complicated, likely defeating the purpose of carrying out a systematic review of this type (Mertz 2019). Therefore we did not conduct a traditional quality appraisal, we instead applied Dixon-Woods et al. (2006) approach, namely that throughout the review we were mindful about the credibility and contribution of the papers that were included. In addition we provide conclusions more generally, about the general strengths and shortcomings of this literature as a whole in our discussion.

### Data Extraction and Synthesis

A data-extraction pro-forma was devised that identified the study, a summary of its major arguments and the major themes that emerged from the paper. Data was synthesized with the aim of creating a “synthesizing argument” in line with the research above.

## Results

In total twenty-four papers were included in the below synthesis. In relation to what codes do, the results have been divided into six sections related to the functions of codes. This includes 1) guidance—the guidance codes offer for health workers, 2) professional status and identity—the relationship between codes and broader professional status and identity, 3) specific structure or content—this deals with arguments about the structure or content of codes, 4) theoretical grounding and development of codes—this deals with the development of codes and their theoretical grounding, 5) regulation or enforcement—this includes concerns over the enforceability of codes and 6) the social function of codes—including how codes convey meaning to the public and their broader social benefits.

### Guidance

How and whether codes provide guidance to health professionals is arguably the issue that most dominates the literature. One point of consensus was that codes of ethics functioned to provide guidance to those who belong to the profession in question. This is often explicitly acknowledged in codes themselves. A number of codes contain guidance about their interpretation and their limitations. For example, the Internatioal Council of Nurses (2021) code notes that what is presented in the code “are not intended to be an exhaustive or complete list of concepts.” Other codes have been specifically designed to facilitate ethical decision-making, for example the Canadian code for psychologists provides a decision-making model, which contains seven steps to aide ethical decision-making (this will be discussed more below) (Hadjistavropoulos and Malloy 2000). While it is perhaps uncontroversial to say that the majority of codes provide guidance, the extent to which they do this is contested. On this point, it is notable that most criticisms were dependent on the assumptions made by authors as to how specific codes should be in the guidance they provide. Views about guidance often sat on a spectrum. At one end, there was the belief that codes should provide guidance and that such guidance was influential in shaping ethical conduct. At the other end of this spectrum was a far more modest expectation related to codes, namely that, at best, they could only provide general principles that enable and encourage ethical conduct. Obviously if one believes codes should provide detailed and exhaustive guidance, there was far more room for criticism for failing to do so. Criticism in relation to guidance generally fell in to one of four categories, 1) a lack of clarity around concepts or principles, 2) the breadth or specificity of the guidance, 3) conflicting principles or obligations as they relate to the guidance, and 4) the application of guidance in practice. Each of these issues overlap and were somewhat interrelated, so should not be seen isolation; each will be discussed below.

On the first point, related to clarity, reviewing the concept of social justice in U.S. nursing documents (including the code of ethics), Bekemeier and Butterfield (2005, 152) concluded that “these documents present an inconsistent, ambiguous, and superficial conceptualization of social justice, and do not offer an adequate framework for nurses to address underlying issues that affect health outcomes.” They conclude that any nurse who sought advice in relation to social justice would have to undergo “awkward translations of fundamental nursing standards” to identify what types of action may be permissible. Over a decade later, similar issues were noted by Valderama-Wallace (2017, 152) who again examined U.S. nursing documents, finding “ongoing inconsistencies in conceptualizations of social justice.” Similar concerns were raised by Bersoff (1994) in relation to the U.S. psychology code, noting that while the code was improved in that it had “increased breadth and specific attention to important issues not addressed in the previous code” (382) it also had “rampant qualifying language” (382) which often diluted messages within the code. These criticisms have extended to codes more generally. Komparic et al. (2023, 406) argue that codes are inherently interpretive and “multivocal” in that they “usually underdetermine or provide more than one answer to the question.”

The second major issue relates to the specificity or breadth of the guidance within codes. Codes could be criticized either way on this point, that is, if a code were too specific it could become unwieldy in its length and complexity (this would also increase the change on principles or guidance clashing, discussed below). Dahnke (2009, 116) argues for example “any code of ethics is limited by the infinite variety of concrete, particular situations in which any individual nurse is likely to find himself or herself.” There is of course the other problem that such a detailed code simply wouldn’t be possible, namely that, “no code can reliably foresee changes in technology and environmental and organizational structures that might bring new ethical challenges” (Dahnke 2009, 116). On the other hand, codes could also be criticized for being too “broad and unspecific that they are of no practical help, leaving enormous scope for interpretation in specific situations” (Hussey 1996, 253). Snelling (2017, 402) examined the U.K. nursing code and concluded that the “minimalist position” taken by the U.K.’s Nursing and Midwifery Council “on the provision of guidance is unconventional compared with others, leaving registrants unable to ascertain action guiding meaning from ambiguous clauses in the Code and educators unable to use it in teaching.” Obtuse or vague statements could lead to other difficulties, notably that ethically problematic behaviour could still be permissible where guidance is too broad (O’Donohue 2020). Importantly, the issue of breadth and specificity did not necessarily sit on a spectrum, any given passage in a code could be both specific and vague at the same time. Hussey (1996, 253) provides an example from the U.K. nursing and midwifery code, that stated that nurses must “work in a collaborative and cooperative manner with health care professionals and others involved in providing care, and recognize and respect their particular contributions within the care team.” Such a passage, is arguably “both specific to their professional situation and vague about the nature and limits of the commitment” (Hussey 1996, 253).

A further criticism relates to the potential for conflict within codes and between other codes, policy, principles, or even the law to which the healthcare professional had obligations. This has been labelled the “multiplicity problem” elsewhere (Eriksson et al. 2008), that is, that any health professional may be subject to any number of codes, policies, or even laws that may also clash with their code of ethics. O’Donohue (2020) argues, for example, that the U.S. psychologists’ code could be seen to clash with civil rights in the United States, notably the constitutional right of freedom of speech. When faced with conflicts within a code, there is often little in the way of guidance (however some codes have been arranged hierarchically in an attempt to address this, see below).

A final criticism relates to the application of the above guidance, whether this be related to individual fallibility or the difficulty in bridging the gap between theory and practice. Firstly, the interpretation and application of these concepts by health professionals is far more messy in practice, with their interpretation and application dependent on a range of contextual factors (Eriksson et al. 2008). This limitation relates to what Pattison (2001) calls, “ordinary moral experience,” namely that codes “largely ignore the experience and judgement that professionals bring with them from living and developing within the wider moral community that is society” (11). One further notable criticism relates to the fact that there is empirical evidence to suggest that ethical reasoning can differ between the genders, raising a further issue in regards to the potentially gendered nature of codes (O’Donohue 2020). Perhaps more fundamentally, O’Donohue (2020) argues that far too little attention has been given to how people make ethical decisions and what occurs when they engage in unethical behaviour. Behaviour could be the result of “free will/choice … to a complex cognitive decision, to blindly following impulse or intuition with little to no cognitive activity” (281). They go on to argue that there is a need for greater and more “fine-grained” analysis of such decision-making. One final issue related to the application of codes is that an over-reliance on codes could actually lead to unethical behaviour. This is what has been called the “legalisation problem,” (Eriksson et al. 2008) that is by treating codes as akin to legal documents,

… [a]ctions may be formulated in order to minimally meet the perceived requirements of the code rather than toward developing a coherent, comprehensive, and reflective ethical life. Health professionals may lose a sense of accountability if they believe the answers to difficult ethical questions are readily obtainable from a pre-existing document rather than being found through a process employing their own reasoning, conscience, and character. (Dahnke, 2014, 617)

As noted above, criticism of codes were related to what authors often felt codes should be, whether this be provide exhaustive guidance in almost every situation or more general and more modest principles that could shape behaviour. The question naturally follows, what can we reasonably ask of codes in relation to the guidance they provide? For Pattison (2001, 8), many codes “do little to develop or support the active independent critical judgement and discernment that is associated with good moral judgement and, indeed, good professionalism. They may, in fact, be in danger of engendering confusion, passivity, apathy and even immorality.” While also sceptical of their role in decision-making, Snelling (2017, 392) argues that a certain level of clarity is required, notably,

… they must be clear enough so that nurses understand what their professional practice demands of them, so that educators can use the text and associated guidance to facilitate students’ understanding of the nature of professional practice, and so that regulators can use them to police entry and maintenance on a professional register.

In contrast to these positions, Hussey (1996, 253) argues that criticisms of codes assume healthcare professionals make decisions in a “grossly simplistic deontological way, that it consists in the following of a set of absolute rules, the justification of which depends only on its origin: the edict of an authority.” Similarly, the idea that codes alone ensure ethical conduct is labelled as too simplistic by Banks (1998, 29 who notes “although some codes contain detailed rules in some areas, none prescribes action in all situations, nor, indeed, are they intended to.” Similarly, Black (1984, 180) argues that codes should not be applied to promote “blind adherence, but as a system of relationships which may require substantial modification in the light of the character of the people involved, and of the situations in which they find themselves.” In reality healthcare professionals will utilize their professional judgement, peers, and past experience to also make decisions, amongst other factors. This view is consistent with what is put forth in the preface or explanatory information in codes. Dahnke (2009, 116) notes that most codes do not call for an “inflexible monolithic interpretation,” with codes often mandating “a mindful, interpretive application of … standards and the use of personal judgment.” In lieu of viewing codes as documents that provide comprehensive guidance, Banks (1998, 30) suggests we view “codes of ethics as contributing to the enabling or encouragement of ethical conduct,” not as a “tablet of stone laid down by an outside body and valid for all time.” Whether or not a code provides guidance and the extent to which it does this, remains contested.

### Status and Identity

A further theme that emerged related to how codes shape professional status and identity. Codes are seen (by many) as an indispensable part of any profession and have been critical in the transformation of many of the professions as we know them today. One of the most striking examples in the above papers comes from the United States and the first physiotherapy code. Linker (2005) details the development of this code, which is worth discussing at length. In the United States throughout the 1920 s and 30 s, physiotherapy was an emerging discipline. The workforce was primarily made up of women and was formed in response to World War I and the need to rehabilitate soldiers returning from war. The end of the war saw the discipline take several steps to establish itself as a profession, including the establishment of the America Women’s Physical Therapeutic Association, the scheduling of an annual conference (at the same time and place of the American Medical Association) and the drafting of an organizational constitution. This of course created tension with the American Medical Association (AMA) and others. The AMA had already sought to equate physiotherapy with pharmacy, as an arm of medicine and that it could only become a legitimate profession if led by physicians. The drafting and adoption of a code of ethics was essential in carving out physiotherapy as a distinct profession and in keeping the AMA at bay. Linker (2005) notes that the women who drafted the code, which was adopted in 1935, did not see themselves as moral philosophers and that the code was developed with more pragmatic concerns in mind, notably to put the profession on “firmer ground” and avoid further subordination in the “medical chain of command.” What is notable about this code is that it did not contain any statements about the physiotherapist–patient relationship and largely focused on the relationship between physiotherapists and physicians. This was likely a deliberate decision to “overcompensate for the gender make-up” of the discipline and to maintain a “gender neutral” identity. The physiotherapy code carved out a professional identity in sharp contrast to other disciplines, like nursing who did not adopt a formal code until the 1950s. Purvis (2023, 5) argues that nursing codes have historically, “[reflected] a dominant patriarchal hegemony that reinforced the subject position of nurses as loyal helpers carrying out physician orders.” Linker (2005) argues that the development of a code played a substantial role in securing physiotherapies place in the medical profession and this case is illustrative of how codes serve as “statements of distinct fears, concerns, and desires of a professional group of people in a specific time and place.” (323)

The above historical example is one of the most striking when it comes to codes and their role in defining and cementing professional identity and status. This has not gone unrecognized elsewhere, with a number of authors pointing toward more contemporary examples. Hussey (1996, 252) argues that the “very existence of a code proclaims that the group aspires to the status of a profession, with moral respectability and autonomy” and that codes provide a means for the profession to negotiate with others and retain the status to self-regulate. Dahnke (2009) suggests that codes form part of the social contract, between individuals and the profession as a whole; having agreement around a code results in a stronger profession. Notably however and while a code may provide cohesion for professions, professionalism itself and codes have evolved substantially over time. The medical profession has also had an interesting history in this respect. Sox (2007) shows not only how codes were used to establish identity and power for the medical profession in the United States but how this has been eroded over time, with Sox (2007, 1539) concluding that the “code of conduct may soon be the sole vestige of its historical position as a powerful guild.”

### Content and Structure

There has been much said about the specific content and structure of codes. Discussion here ranges from the inclusion or exclusion of specific items in any given code, to how codes are laid out to aide decision-making.

A number of papers spoke about specific issues related to the inclusion or exclusion of specific items or statements, such as spirituality or human rights. However, there were a number of more general concerns raised. Notably codes do not often contain details about reciprocal duties, that is, while healthcare professionals have duties to others, patients, employers, and others do not necessarily have to reciprocate in any way (although these of course could also be included in other documents, O’Donohue 2020). Similarly, codes are often focused on the individual, failing to take into account “ethical matters between individuals and classes of people or between multiple classes” (O’Donohue 2020, 280). Related to these critiques, namely on the information that codes include, many codes often provide a mix of content, from ethical guidance, to guidance about quality, and other matters (something which will be discussed below).

Interestingly, a number of codes have been arranged specifically to aide decision-making, to assist professionals in charting the most ethical course of action. For example, the U.S. nursing code contained nine provisions, the first three of which describe the most pressing values and commitments. While not explicit, “the division and categorization of the provisions suggest a hierarchy in which the duties outlined in the first 3 provisions take precedence” (Dahnke 2009, 115). While structuring codes has been an attempt to overcome some of their limitations and critiques, this often raises even more questions. Williams (2004, 26) for example suggests that the model of ethical decision making in the New Zealand code for psychologists, “demands cognitively explicit, linear, rational decision-making” which fails to take into account the evidence on how ethical decisions are made. Similarly, O’Donohue (2020) argues in relation to the U.S. psychology code, that the codes is presented in “an ex cathedra, irrationalist manner,” noting that for any of the statements in the code, one could ask “Why?—what is the argument or evidence for this assertion?” (281). This last point ties in to the issues raised below when considering the foundations of codes, the foundations of their authority, including their theoretical grounding and development.

### Theoretical Grounding and Development

What meta-ethical or normative assumptions do codes make and from where do they derive their authority? These more fundamental questions are what could be called the foundational problems of codes. On the first question, many codes are often not explicit about their meta-ethical assumptions or commitments. This poses a problem for codes on a number of fronts, related to decision making and from where they derive their authority. O’Donohue (2020, 277) argues that the U.S. psychology code, “is silent on all these important meta-ethical questions.” Similarly, Pattison (2001, 9) argues “most codes do adopt at least some ‘high’ universal moral principles that would be widely recognized and accepted as such. However, these principles are given equal status with precepts that cannot be derived so directly from universal ethics.”

When it comes to normative ethical commitments, the importance of codes having an underlying theory is emphasized by Eriksson et al. (2008) who argue that an underlying theory is critical to enable the application and interpretation of a code. Ballou and Bryant (1997) demonstrate the importance of the theoretical underpinnings of codes, arguing that a feminist grounding of nursing codes could better highlight the role of nurses in promoting healthcare for the good of society, that is, re-focus codes on systematic and structural issues. In their own words, a feminist code would state explicitly “that nurses are expected to engage actively in social action toward improving institutions, national policies, and the ability of the nursing profession to function in a self-defined ethically responsible manner” (82). They argue that such a code would provide nurses greater scope in addressing systems that limit their ability to provide such care. Similarly, Thompson (2002) argues that ethics as they relate to midwifery should be grounded in feminist-relational ethics, notably “[i]f midwifery is a shared tradition of woman and midwife then the ethic of midwifery practice ought to be one based on a consensus of their beliefs and goals rather than on the beliefs and goals of other practices such as science, medicine and moral philosophy” (531).

This raises further issues about authority, even where codes have made their meta-ethical or normative commitments explicit. This is outlined by Hussey (1996) who argues that “[t]he ultimate justification of moral principles is perhaps the most important, profound and difficult issue in moral philosophy” (254). They go on to note that pointing to a code’s origins is not enough: “it has been widely accepted amongst philosophers that moral principles cannot be justified by pointing to their origin, even when this is reputed to be God or the UKCC” (254). Perhaps more pragmatically however, codes are rarely transparent when it comes to their development or who was involved (O’Donohue 2020), there is nothing approaching what might be considered a “best practice” in the development of codes. Even then there remains the issue of fallibility, notably that,

… [a]s much presumptive authority as we might give the considered judgment of those who write and revise these codes; we cannot presume infallibility on their part. Humans, technically and morally, are fallible creatures. As such, we cannot presume any statement on morality—particularly one as complex as a professional code of ethics—to be without flaw, complete, and comprehensive. (Dahnke 2009, 115–116

### Regulation and Enforcement

One point of difference in relation to codes is whether they are designed to provide largely aspirational standards or to regulate and enforce behaviour. Snelling (2016) argues that codes can be distinguished between their aspirational and regulatory functions, noting that regulatory elements of a code function in a quasi-legal way, namely they are enforced when behaviour violates a specified standard. It is notable, that this is often the distinction between codes of conduct and codes of ethics, however in reality many codes integrate both aspirational and regulatory functions. While Snelling (2016) argues that some codes successfully integrate these functions, others do not, specifically identifying the U.K. nursing codes as conflating both of these functions, creating ambiguous guidance. O’Donohue (2020) argues that there is no clear distinction between codes that are enforceable and those that are aspirational and that we cannot necessarily demarcate ethical behaviour that is aspirational and that which is mandatory. Similarly Pattison (2001) argues that

“there is a great deal of difference between a document that aims to support the emergence of independent, ‘ethical’ professionals … and one that aims to provide clear rules for action that form the basis of professional conformity and discipline” (8).

They go on to note that to label such codes as “ethical” is also potentially misleading.

A related question is whether a code should be enforced at all. Elsewhere this has been called the futility problem, namely that an ethical health professional should not need a code; an unethical health professional will ignore or overlook a code. The need for enforcement of the provisions within a code was assumed by a number of authors. In relation to codes of conduct, Hussey (1996, 252) asserts that a code of conduct “allows transgressions to be identified and justifies penalties, thus becoming an instrument of the authority of a governing body, where such exists.” O’Donohue (2020) asserts that it is naïve to see codes as ethical documents, arguing that when included in licencing laws, behaviour can be both unethical and illegal.

Several further issues present themselves in relation to enforcement, assuming this is part of the function of codes. Codes often contain little information about the adjudication of complaints and even if they do, there is no guarantee that complaints will be adjudicated consistently and fairly (O’Donohue 2020). A further issue arises when considering the reporting of complaints, namely that unethical behaviour is unlikely to be detected (O’Donohue 2020) and that any enforcement would be particularly difficult (Dahnke 2014).

### Social

Beyond what has been discussed above, codes serve several other purposes and for parties outside of the profession, these functions are what could be labelled the social functions of codes.

One issue that has received attention relates to codes function to inform the public of the standards that should be expected of the profession, thus protecting the public. This was seen to have a mutual benefit for the profession, notably that a code “tells clients, colleagues, employers and society what standards to expect, so promoting confidence and trust” (Hussey 1996, 252). The issue of guidance however again was questioned here, namely that if a code had broad or vague principles, it may not only impact the interpretation of the code by healthcare professionals but it may also provide little information to the public and other third parties (O’Donohue 2020). Related to this point, “codes assume a consensus on values that may no longer exist in our increasingly pluralistic, multicultural society” (Hussey 1996, 255). Questions about how to balance such pluralistic values alongside the values of the profession in question are often not discussed or made explicit in codes.

One paper that stands in contrast, offers a relatively optimistic assessment of the AMA code of ethics (Baker and Emanuel 2000). While the authors acknowledge that the AMA was “also involved in other activities that seem less noble-minded” (15) the authors contend that the AMA also played a role in addressing “fee-splitting” (i.e. referrals to others for a commission), promoting scientific research by opposing anti-vivisection laws and having a hand in the establishment of the U.S. Food and Drug Administration. The authors argue this provides evidence that the AMA was working for the U.S. public and putting their interests first, working systematically on these issues even though they did not serve their financial interests. They attribute these actions, at least partially, to the code adopted by the AMA. Other papers however were more sceptical in regards to the role of codes in social and policy change. Komparic et al. (2023) for example argue that codes are particularly poor instruments to promote policy change as they are inherently “multivocal” that is, “usually underdetermine or provide more than one answer to the question” (406). Critical views can be found elsewhere, Bekemeier and Butterfield (2005) argue that U.S. nursing documents (including the code) “fall short of espousing clear, consistent direction to nurses in the United States who participate in social reform or who seek guidance in what is expected of them” (161). Importantly it was not just codes (lack of) influence on broader society. Teo (2015, 78) argues that the U.S. psychology codes were “not immune to ideological changes” that occurred in the United States after the September 11 attacks, with the codes shifting from “a principled approach to an approach that seems to emphasize pragmatic common sense and legal authority” which in part had little to say about psychologists participating in torture such as waterboarding. Notably, others have similarly noted how, “[p]olitical influences, philosophical trends and the conceptualization of human rights have shifted how human rights are taken up” within codes (Tisdale and Symenuk 2020, 1085).

## Discussion

This paper sought to critically explore the function of codes of ethics related to the healthcare professions. So, what do codes do? The short answer to this is, from a theoretical perspective, quite a lot. According to the literature, codes fulfil multiple purposes, from providing guidance on ethical issues, to assertions about acceptable and unacceptable behaviour. It has also been argued they are influential in regards to the status and identity of the professions. They vary substantially in their content and structure, their meta-ethical assumptions, and from where they draw their authority. Some codes are aspirational and some are enforceable; most codes contain a mix of things however, with varying success to how these are integrated. Finally, a number of authors contend that codes also serve a number of social purposes. While they undoubtedly convey information to others outside the profession, their role in broader social change is more contested and there are a number of examples of codes that have been influenced by broader social and political forces, often not for the better. The extent to which a code does each of these things varies substantially. That is, the interesting thing is not that any one code does any one of these things but how these are balanced within codes and how forthright codes are when it comes to their function. This review cannot of course answer this question (see below).

The above criticisms should not be seen in isolation. A code that is brief and written in broad terms, will serve a different purpose to those that are lengthy with detailed rules, each will also come with different trade-offs in the information they convey to professionals and those outside the profession. A code that is aspirational will also come with trade-offs when compared to those which are regulatory. The complexity here is furthered when we think about the other considerations related to codes that have yet to be touched on above, in how codes fulfil their functions. Banks (1998) suggests that codes do this in several ways through their content which could fall into one of four categories: 1) ethical principles—general statements that underpin how a professional should behave, such as respect for autonomy or promoting justice, for example; 2) ethical rules—more direct statements that provide direction on what *must* be done or *avoided* for example; 3) practice principles—general statements about achieving what is desired, for example teamwork, collaboration, or communication; 4) practice rules—specific statements about practice, for example the prohibition of advertising for example. Codes could of course contain any combination of the above principles and rules and could of course fulfil any or all of the functions discussed throughout this paper.

This raises a broader and arguably more fundamental question that has only been discussed in passing above, namely, what is a code of ethics? In terms of normative analysis, this presents a challenge, we first need to define what a code *should* do before we assess its shortcomings. If the primary purpose of a code of ethics is to provide guidance, many fail here, the devil is in the detail however. To what extent should codes provide guidance? As was noted in the above discussion, the functions of codes and the criticisms that often follow are dependent on what we think codes should do/be.

More pragmatically, codes of ethics clearly are not going anywhere. They clearly serve a number of purposes for the healthcare professions, even if all they do is outline general expectations and assert professional authority, codes will clearly remain a feature of the healthcare landscape for years to come. Codes however are not the only answer. Davis (2003) contends that codes are a much more recent phenomenon than commonly thought and that “alternatives to professional codes may help us to understand better what a profession offers its members (and why some governments encourage professions and others do not)” (451). Three decades ago, Dawson (1994) called for a cognitivist account of ethics as a means to address the shortcomings of codes, notably in assisting healthcare professionals to be ethically sensitive and responsive to ethical conflicts that would otherwise not be addressed in codes.

There are a number of limitations with the above review worth mentioning. Notably, this review did not include any papers that explored the nature of codes and the impact of culture and only a few explored the historical developments of codes. Thus, this review says little about historical realities such as colonization and the role of codes here. Because of the nature of the review, we cannot say definitively that such work does not exist, however this does seem like an important issue that should be taken up in future research. Secondly, this review also does not take into account the empirical literature. While scoping reviews exist, further work and synthesis on comparative studies about codes could be particularly fruitful in adding depth to the findings presented here. Finally, this review did not review codes of ethics themselves or related regulatory documents. This would be a huge undertaking to examine potentially hundreds of codes, their content, and function. While some recent studies have attempted to do this (Essex, et al. 2024), there appears to be scope to do far more when it comes to the synthesis of codes themselves to better understand their functions, similarities, and differences.

Bersoff (1994) contends that codes will inevitably be “anachronistic, conservative, protective of its members, the product of political compromise, restricted in its scope, and too often unable to provide clear-cut solutions to ambiguous professional predicaments”. While we share this sentiment, we are also pragmatic and feel that discussions about codes should continue as they are likely to remain a feature of the healthcare professions into the foreseeable future. Perhaps what is needed however is a more candid discussion around the functions and limitations of codes in the first instance and the assumptions that we make in criticizing them. We should also look beyond codes for guidance (if that is of course what codes do), to ourselves and to other accounts of ethics and ethical behaviour.

## Data Availability

Data for this paper is publicly available.

## Footnotes

1 The extent to which codes are ‘important’ for individual health professionals and professional associations will of course differ and is something that we do not discuss here.

2 While we acknowledge that codes of conduct and codes of ethics are not the same, nor do they always serve the same function, for the most part there is often substantial overlap. For this reason, rather than limit our search and analysis, we opted to include codes of conduct.
